# Huangqi Guizhi Wuwu decoction promotes M2 microglia polarization and synaptic plasticity via Sirt1/NF-κB/NLRP3 pathway in MCAO rats

**DOI:** 10.18632/aging.204989

**Published:** 2023-08-30

**Authors:** Zhijie Ou, Min Zhao, Ying Xu, Yan Wu, Lina Qin, Li Fang, Hong Xu, Juping Chen

**Affiliations:** 1 Department of Neurology, Changshu Hospital Affiliated to Nanjing University of Chinese Medicine, Changshu 215500, Jiangsu, China; 2 School of Chinese Medicine and School of Integrated Chinese and Western Medicine, Nanjing University of Chinese Medicine, Nanjing 210023, Jiangsu, China

**Keywords:** Huangqi Guizhi Wuwu decoction, microglial polarization, neuronal plasticity, stroke, Sirt1/NLRP3

## Abstract

Huangqi Guizhi Wuwu decoction (HGWD) has been demonstrated to ameliorate cerebral ischemia-reperfusion injury in clinical application. Nevertheless, the exact mechanisms of HGWD have not been conclusively elucidated. This study aimed to investigate the potential role and mechanism of HGWD on neurological deficits in a rat model of middle cerebral artery occlusion (MCAO). Our results showed that HGWD significantly alleviated neurological deficits in MCAO rats, evidenced by high mNSS score, reduced cerebral infarction area, and improved brain pathological injury. Besides, HGWD reduced the levels of TNF-α, IL-1β, IL-6, SOD, MDA and GSH in the brain tissue. Further study suggested that HGWD promoted microglia polarization towards M2 by inhibiting M1 activation (Iba1^+^/CD16^+^, iNOS) and enhancing M2 activation (Iba1^+^/CD206^+^, Arg-1). Additionally, HGWD increased dendritic spine density and enhanced levels of synapse marker proteins (PSD95, Synapsin I). HGWD also up-regulated Sirt1 expression while inhibited p-NF-κB, NLRP3, ASC, and cleaved caspase-1 level in the hippocampus of MCAO rats. Sirt1 specific inhibitor EX527 notably weakened the neuroprotective efficacy of HGWD against cerebral ischemia, and significantly abolished its modulation on microglia polarization and synaptic plasticity *in vivo*. Collectively, our findings suggested that HGWD ameliorated neuronal injury in ischemic stroke by modulating M2 microglia polarization and synaptic plasticity, at least partially, via regulating Sirt1/NF-κB/NLRP3 pathway, further supporting HGWD as a potential therapy for neuroprotection after ischemic stroke.

## INTRODUCTION

Ischemic stroke, also known as “stroke”, is a serious disease with a high rate of disability and death worldwide, posing a serious threat to human life and health. The China Stroke Report 2019 shows that stroke mortality in China can reach 149.49/100,000 in 2018, accounting for 22.33% of the total mortality of Chinese residents and ranking as the third leading cause of death, only below malignant tumors and heart diseases [1]. Stroke can be divided into ischemic stroke and hemorrhagic stroke. In patients with ischemic stroke within the treatment time window, the best treatment modality is revascularization, including pharmacologic and mechanical recanalization [2]. Many studies have confirmed that revascularization is the most effective treatment modality for ischemic stroke. However, revascularization is prone to vascular neurological impairment and affects patient prognosis [3]. Therefore, there is a need to find safe and effective neuroprotective and reparative treatment modalities to help patients improve their prognosis.

Neuroinflammatory response after stroke plays an important role in regulating the survival of neurons, and inflammation regulation is the key to the treatment of stroke [4, 5]. Microglia are resident innate immune cells in the brain [6, 7]. Neuroinflammatory response is closely related to the activation and polarization of microglia in the brain [8]. After cerebral ischemia and hypoxia, microglia are rapidly activated, and the activated microglia differentiate into different phenotypes and regulate central nervous system homeostasis in different ways [9]. M1 phenotype, also known as classical activated microglia, further aggravates cerebral ischemic injury by releasing different proinflammatory mediators. The M2 phenotype, alternatively activated, can improve local inflammation and is essential for tissue preservation and brain repair [10]. In-depth study of the transformation mechanism of different microglia phenotypes, promoting the transformation from proinflammatory phenotype to anti-inflammatory phenotype, can be used as a new treatment strategy for stroke, help to better select the target of action, which is conducive to the treatment of the disease. However, the underlying mechanisms of how microglia change their phenotype after ischemia are largely unknown.

NLRP3 inflammasome is a key mediator of inflammatory response after stroke. Typically, NLRP3 inflammasome becomes self-oligomerization once activation, recruits and activate pro-caspase-1 with the assistance of connexin-ASC to form a new molecular platform, called NLRP3 inflammatory complex [11]. NLRP3 inflammatory complex can shear inactive pro-caspase-1 to active caspase-1, activate proinflammatory factors, form inflammatory reaction waterfalls through autocrine and paracrine ways, and release a large number of cytokines and chemokines [12]. Besides, NLRP3 can mediate the transition of microglia from resting state to activated state, and aggravate cerebral ischemia-reperfusion injury in mice by inducing neuronal apoptosis and promoting the release of inflammatory factors [13, 14]. NLRP3 could be activated by proinflammatory gene NF-κB [15]. Besides, sirtuin 1 (Sirt1), an NAD+-dependent deacetylase, has been shown to negatively regulate NLRP3 activation and thereby inhibit the inflammatory response [16].

Traditional Chinese medicine has rich practical experience and potential advantages in treating stroke. Huangqi Guizhi Wuwu Decoction (HGWD), derived from the Golden Chamber Synopsis, is a classical formula prescribed for treating blood stasis. HGWD includes five herbs, namely Radix Astragali, Ramulus Cinnamomi, Paeoniae Radix Alba, Zingiberis Rhizoma Recens, and Jujubae Fructus*.* According to modern clinical studies, HGWD exerts multiple neuroprotective effect in ischemic stroke, diabetic peripheral neuropathy as well as oxaliplatin induced peripheral neurotoxicity [17–19]. HGWD can significantly improve the neurological functions of stroke patients and improve cerebral blood flow [20]. HGWD contains a variety of neuroprotective components against cerebral ischemia injury, such as paeoniflorin, calycosin glycosides and calycosin. Paeoniflorin was reported to regulate Ca2+/CaMKII/CREB signaling to alleviate cerebral ischemia-reperfusion injury [21]. Calycosin glycosides can attenuates neuronal damage by preventing oxidative stress via the Sirt1/FOXO1/PGC-1α Pathway in HT22 cells [22]. Calycosin preserved BDNF/TrkB signaling and reduces post-stroke neurological injury after cerebral ischemia by reducing accumulation of hypertrophic and TNF-α-containing microglia in rats [23]. However, the mechanism of HGWD in improving cerebral ischemic injury and its effect on microglial cell polarization remain unclear. In this study, the neuroprotective effect and underlying mechanism of HGWD were investigated using MCAO rat model.

## MATERIALS AND METHODS

### Reagents

Crude herbs including Radix astragali, Ramulus cinnamomi, Radix paeoniae alba, Rhizoma zingiberis recens and Fructus jujubae, were all purchased from Puning Zequn Chinese Herbal Pieces Co., Ltd. Trizol and BCA assay reagent were both purchased from Beyotime Biotechnology (China). PrimeScript™RT reagent Kit and TB Green ® Premix Ex Taq™ II (TliRNaseH Plus) are purchased from TaKaRa. Antibodies against SIRT1, NLRP3, ASC, PSD-95, Synapsin-1, Iba1, CD16, CD206, p-NF-κB, GAPDH, anti-rabbit IgG (H+L) and Anti-mouse IgG (H+L) were bought from Cell Signaling Technology (Danvers, MA, USA). Cleaved caspase-1 was purchased from Abcam (Cambridge, UK). EX527, a SIRT1 inhibitor, was bought from Sigma-Aldrich (St. Louis, MO, USA).

### HGWD preparation

Radix astragali 90 g, Ramulus cinnamomi 90 g, Radix paeoniae alba 90 g, Rhizoma zingiberis recens 180 g, and Fructus jujubae 90 g were weighed. The above herbs were soaked in water (8 times the total weight) and refluxed twice, 1 h each time. The liquid extracts were filtered, combined and concentrated to 100 mL by a rotary evaporator. The extract were the freeze-dried to powder (59.9 g), which were stored at −20° C.

### Quality control analysis for HGWD extract

HGWD extract was analyzed by using high-performance liquid chromatography system (HPLC) in which paeoniflorin, calycosin-7-O-β-D-glucoside, ononin, calycosin, cinnamic acid, and formononetin were used as quantitative stands. HGWD power was dissolved in methanol, and then filtrated with 0.22 μm filter. HGWD solution (10 μL) was injected into HPLC, and separated at a flow rate of 1.0 mL/min with a Waters sunfire-C18 column (250 mm × 4.6mm, 5 μm). The mobile phase consisted of acetonitrile (A) and 0.1% formic acid (v/v) (B). The separation temperature was 30° C, with UV detection performed at 260 nm.

### Animals

Sixty healthy male Sprague-Dawley rats (8 weeks old, weighing 290~310g) were purchased from SPF (Beijing) Biotechnology Co., Ltd. [license No. SCXK (Beijing) 2019-0010)]. This research scheme was approved by the Animal Ethics Committee of Nanjing University of Chinese Medicine. All rats were housed at 25° C with 60% relative humidity and 12-h light/dark cycle. Standard rodent chow diet and water were available. All research scheme was approved by the Animal Ethics Committee of Nanjing University of Chinese Medicine.

### Middle cerebral artery occlusion (MCAO) surgery

The animals were randomly divided into 5 groups: the control group (the sham operation group), model group (the MCAO group), HGWD low-dose group (0.38 g /kg), HGWD high-dose group (0.76g/kg), and edaravone group (4 mg/kg). MCAO surgery was performed according to Longa’s method. Rats were anesthetized by 2-3% isoflurane. Then the right common carotid artery, external carotid artery and internal carotid artery were separated successively. The main external carotid artery was ligated and dissociated. A small incision was made in the external carotid artery, and the nylon cord (0.28mm) was gently inserted into the internal carotid artery to block the middle cerebral artery. After 90 mins, the nylon cord was gently pulled out to restore blood perfusion. Ligate the external carotid artery and suture the skin. The middle cerebral artery was not blocked in the sham operation group. Twenty-four hours after ischemia, rats in the HGWD groups were intragastrically administered HGWD extract, and the edaravone group was intraperitoneally injected for 7 days. The sham group and model group were given the same amount of normal saline by gavage.

### Neurological evaluation

The neurological deficit of rats was evaluated according to the modified neurological severity score (mNSS) criteria [24]: 0, no deficit; 1-6, mild deficit; 7-12, moderate deficit; 13-18, severe deficit. Neurological severity score (mNSS) of rats was performed at at 24 h, 3 d, and 7 d after the MCAO surgery, by researcher who was blind to the drug treatment.

### 2, 3, 5-triphenyltetrazolium chloride (TTC) staining

At the end of the experiment, rats were thoroughly perfused with saline under deep anesthesia. Then the brains were quickly removed and frozen at -20° C. The brain was sectioned coronally into 2mm slices, which were incubated with 1%TTC for 10 min in the dark. After staining, the un-infarcted part in the brain visualized to be red, while the infarcted tissue was white. After taking pictures, the Image J software was used to analyze the area of cerebral infarction.

### Hematoxylin-eosin (HE) staining

The collected brain tissue was fixed with 4% paraformaldehyde, embedded in paraffin, and sliced into sections with a thickness of 5 μm. After routine dewaxing to water, HE staining was performed, and histopathological changes of brain tissues of each group were observed under an optical microscope (Olympus Optical Ltd., Tokyo, Japan).

### Oxidative stress and inflammatory cytokines detection

MDA, SOD and GSH levels in the brain tissues were measured with their commercial assay kits (Nanjing Jiancheng, Nanjing, China). The levels of tumor necrosis factor-α (TNF-α), interleukin-6 (IL-6) and IL-1β in brain tissue were determined by enzyme-linked immunosorbent assay.

### RT-PCR to detect CD16, iNOS, CD206 and Arg-1 mRNA expression in hippocampus

Total RNA was extracted from the brain tissues by TRIzol reagent and reversely transcribed into cDNA according to the instructions of reverse transcription kit. The mRNA level of CD16, iNOS, CD206 and Arg-1 were determined by qRT-PCR. The reaction conditions were set as follows: pre-denaturation at 95° C for 5 min, denaturation 95° C for 10 s, and annealing under 60° C for 30 s, 45 cycles. The level of CD16, iNOS, CD206 and Arg-1 was quantitatively analyzed by 2-ΔΔCT. The primers used were as follows: CD16: forward, 5’-TCCGTGGCAGTCTATGAGGA-3’, and reverse, 5’-CAGATGGTGAGGTCGCAAGT-3’; iNOS: forward, 5’-AGTCAACTA CAAGCCCCACG-3’, and reverse, 5’GCAGCTTGTCCAGGGATTCT-3’; CD206: forward, 5’-GGTTCCGGTTTGTGGAGCAG-3’, and reverse, 5’-TCCGTTT GCATTGCCCAGTA-3’; Arg-1: forward, 5’-TCCTTAGAGATTATCGGAGCG-3’, and reverse, 5’-GTCTT TGGCAGATATGCAGG-3’; GAPDH: forward, 5’-GCCAAGGCTGTGGGCAAGGT-3’, and reverse, 5’-TCTCCAGGCGGC ACGTCAGA-3’.

### Immunofluorescence staining to examine the expression of Iba1, CD16 and CD206 in hippocampus

After cardiac perfusion, the brain tissue was rapidly removed with cold PBS, and then embedded in Tissue Tek OCT (Sakura Finetek, Torrance, CA, USA), sectioned into slices (7 μm thickness). Immunofluorescence staining was performed to detect microglia polarization. In brief, the sections were blocked with 1%BSA and 0.3%Triton X-100 at room temperature for 1 h, and incubated with primary antibodies including Iba1 (1:500), iNOS (1:500), Arg1 (1:500), CD206 (1:500) overnight at 4° C. Then, slides were washed with PBS, followed by incubation with Goat Anti-mouse labeled Alex Flour ®488 or Goat Anti-Rabbit IgG labeled Alex Flour ®594 (1:000). After sealing with DAPI reagent, the slides were observed and photographed using fluorescence microscopy (BX63, Olympus Optical, Ltd., Tokyo, Japan).

### Western blotting

Brain tissue protein was extracted using RIPA lysis buffer. The protein concentration was detected by BCA kit. Protein samples were separated by SDS-PAGE electrophoresis, transferred to 0.22 μm PVDF membrane, and blocked with 5% non-fat milk. Then the membrane was incubated with primary antibodies overnight at 4° C. The membrane was washed with TBST, incubated with the secondary antibody IgG (1:10000) at room temperature for 1.5 h. After washing, the brands were visualized with the ECL chromogenic agent. The gray value of each protein was analyzed by Image J software.

### Statistical analysis

All statistical analyses were performed using Graphpad Prism 8 software. Experimental data are expressed as mean±SD. One-way analysis of variance was used for comparison between groups. *P* < 0.05 suggest the difference was statistically significant.

### Availability of data and material

The authors hereby declare that the data and materials in this study will be presented upon request from the corresponding author.

## RESULTS

### Quantitative analysis of HGWD extract

**Figure 1:**
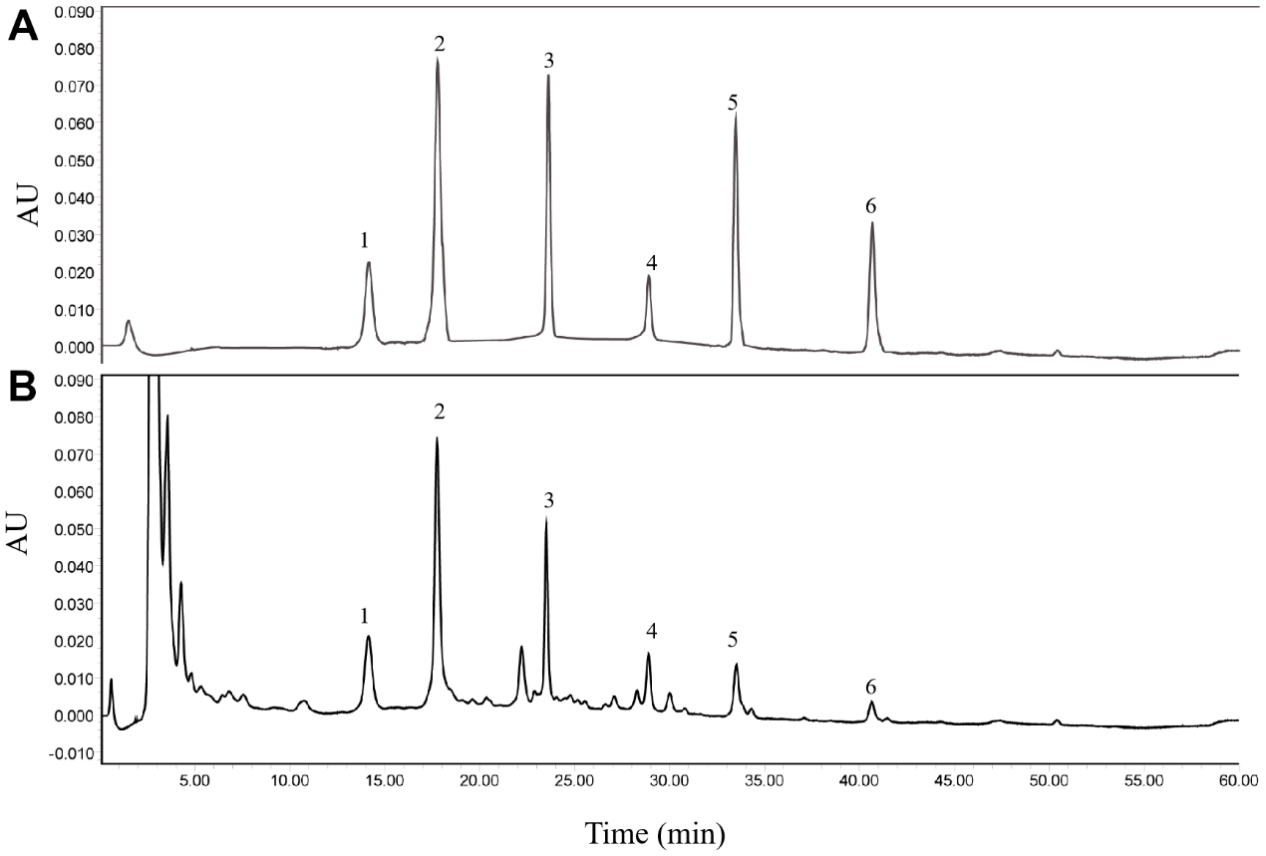
**Representative chromatograms obtained by HPLC-UV.** (**A**) standard compounds. (**B**) HGWD extract. 1, paeoniflorin; 2, calycosin-7-O-β-D-glucoside; 3, ononin; 4, calycosin; 5, cinnamic acid; 6, formononetin.

Firstly, six major ingredients in HGWD extract were identified for the quality control using HPLC-UV method, including paeoniflorin, calycosin-7-O-β-D-glucoside, ononin, calycosin, cinnamic acid, and formononetin. The chromatographic condition was optimized, and representative chromatograms of standard compounds and HGWD extract was presented in Figure 1A, 1B. With the validated HPLC-UV method, the concentrations of paeoniflorin, calycosin-7-O-β-D-glucoside, ononin, calycosin, cinnamic acid, and formononetin were determined to be 1.9728, 0.1134, 0.0741, 0.0129, 0.0325, and 0.0056 μg/mg, respectively, in HGWD extract.

### HGWD protects against brain damage after MCAO in rats

**Figure 2:**
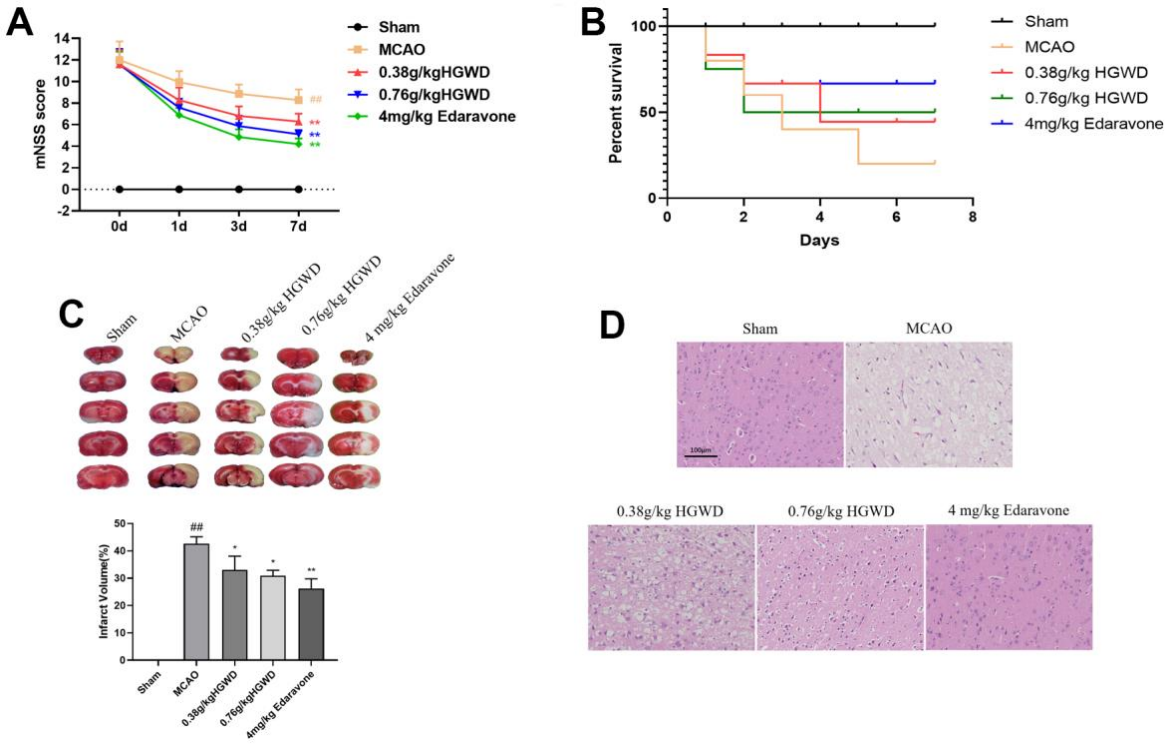
**HGWD improved neurological deficits in MCAO rats.** (**A**) mNSS score. (**B**) Survival rate. (**C**) Infarct size observed by TTC staining. (**D**) Histopathological changes in the ischemic portion of the rat brain observed by HE staining (scale bar= 100 μm). ^##^*P* < 0.01 vs. control group; *P< 0.05, **P< 0.01 vs. model group.

Next, we determined the neuroprotective effects of HGWD *in vivo*. MCAO model rats were established, and modified Neurological Severity Score (mNSS) was applied to evaluate the neurological behavior of rats at day 1, 3, and 7 after MCAO. As presented in Figure 2A, 2B, rat in the MCAO group showed the highest mNSS scores and low survival rate, while HGWD and edaravone treatment significantly attenuated the neurological deficits in MCAO rat. Meanwhile, MCAO/R performance caused notably increased infarct volume according to the TTC staining (Figure 2C). A significant reduction of infarct volume was observed in rats administrated with HGWD at doses of 0.38 and 0.76 g/kg, from 42.65 ± 2.48% to 33.08 ± 4.96%, and from 42.65 ± 2.48% to 30.95 ± 1.95%, respectively, when compared with the MCAO model group (Figure 2C). Besides, we examined the histopathological changes in the ischemic brain section by HE staining. The results showed that HGWD exhibited a protective effect against tissue injury, characterized by less degeneration and necrosis of nerve cells (Figure 2D). These above data suggest that HGWD can relieve cerebral injury induced by MCAO.

### HGWD decreased inflammatory and oxidative stress responses in MCAO rats

**Figure 3:**
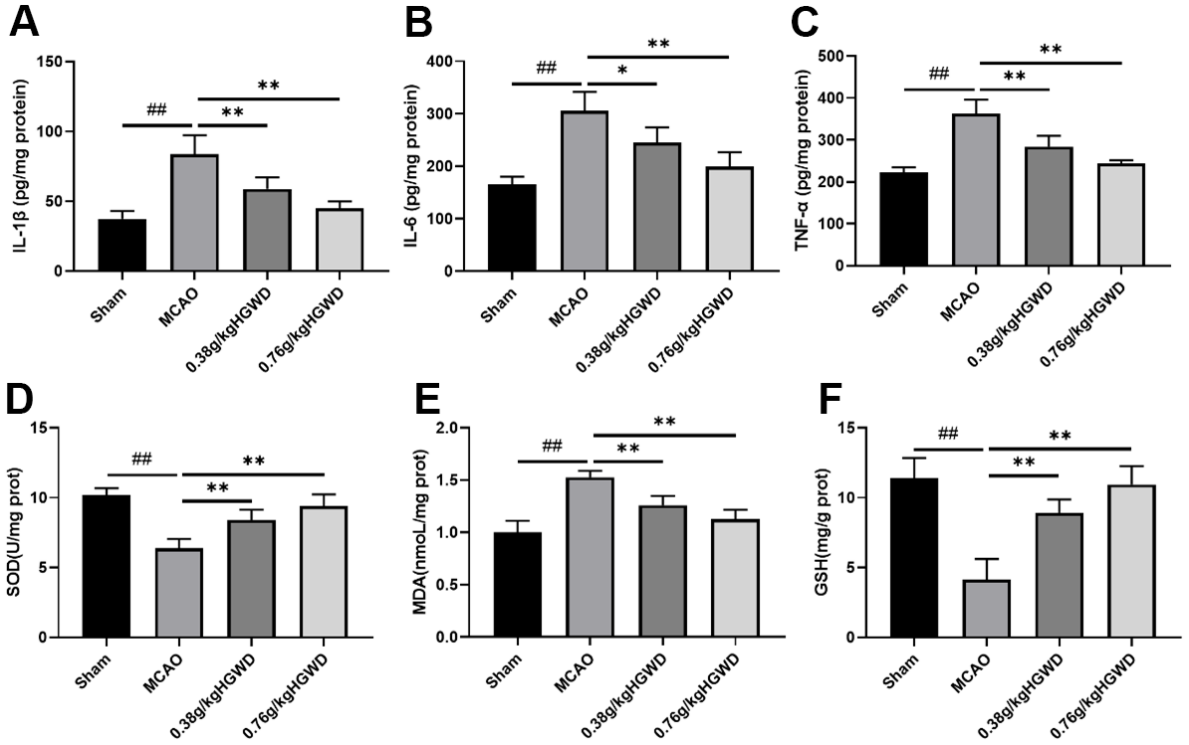
**Effect of HGWD treatment on inflammatory cytokines and oxidative stress responses in brain tissue of rats.** (**A**–**C**) The levels of IL-1β, IL-6, and TNF-α. (**D**–**F**) oxidative stress factors SOD, MDA, and GSH levels in the brain tissue. ^##^*P* < 0.01 vs. control group; *P< 0.05, **P< 0.01 vs. model group.

To understand the effects of HGWD on inflammatory response, we measured the levels of inflammatory cytokines such as IL-6, TNF-α, and IL-1β using ELISA kits. The results displayed that, compared with the sham group, MCAO can distinctly enhanced the release of TNF-α, IL-1β, IL-6 in the brain. HGWD treatment can obliviously inhibit inflammation in MCAO rat (Figure 3A–3C). Additionally, we investigated the effect of HGWD on the oxidative stress mediators, including SOD, MDA and GSH in the brain tissue. As Figure 3D–3F displayed, MCAO rats presented reduced SOD and GSH levels while promoted MDA level. However, HGWD at doses of 0.38 and 0.76 g/kg decreased the elevation of MDA and enhanced SOD and GSH levels. These data indicated that HGWD could weaken inflammatory and oxidative stress responses in rats with MCAO.

### HGWD shifts microglia polarization towards M2 in MCAO rats

**Figure 4:**
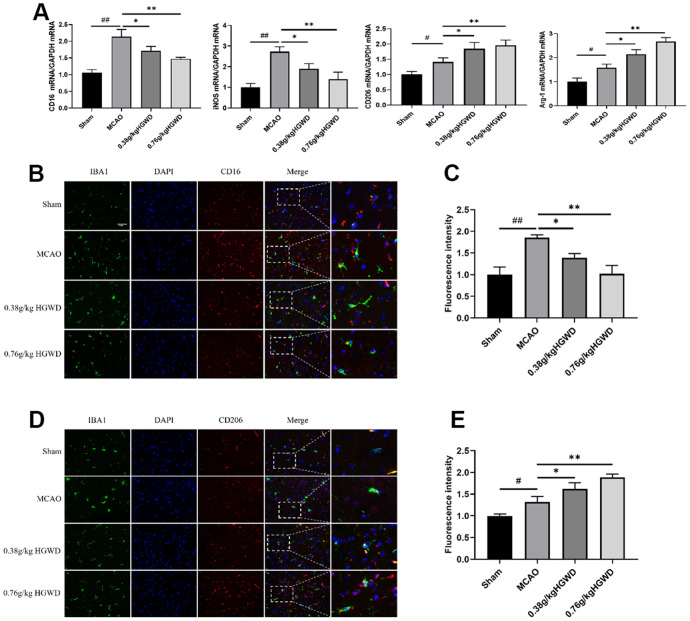
**HGWD promotes the M2 phenotype in microglia after ischemic stroke.** (**A**) M1 marker (CD16 and iNOS) mRNA and M2 marker (CD206 and Arg-1) mRNA levels. (**B**) Representative double-immunofluorescence staining for CD16 (red) and Iba-1 (green) markers in hippocampus. (**C**) Quantification of the fluorescence intensity of CD16^+^/Iba-1^+^ cells. (**D**) Representative double-immunofluorescence staining for CD206 (red) and Iba-1 (green) markers in hippocampus. (**E**) Quantification of fluorescence intensity of CD206^+^/Iba-1^+^ cells. Scale bar: 50 μm. ^#^*P*< 0.05, ^##^*P* < 0.01 vs. control group; *P< 0.05, **P< 0.01 vs. model group.

Many evidences have highlighted the important role of microglia M1/M2 polarization in the progression of inflammation in cerebral injury. Therefore, we investigated whether the neuroprotective effects of HGWD were associated with altered microglia polarization. qRT-PCR experiments revealed the elevated expression of M1-like markers (CD16 and iNOS) and M2-like markers (CD206 and Arg-1) in the ischemic brain of MCAO rats (Figure 4A). However, HGWD could shifted microglia polarization towards M2 phenotype, decreasing CD16 and iNOS expression but increasing CD206 and Arg-1 expression, compared with MCAO model group. Double immunostaining further confirmed that the number of CD16+ M1 microglia and CD206+ M2 microglia were both significantly increased in the brain of rat after MCAO (Figure 4B, 4C). Consistent with qRT-PCR results, HGWD could decreased number of CD16+ M1 microglia while increased CD206+ M2 microglia, suggesting that HGWD promoted M2 polarization in MCAO rats.

### HGWD increased dendritic spine density and enhanced synaptic plasticity in MCAO rats

**Figure 5:**
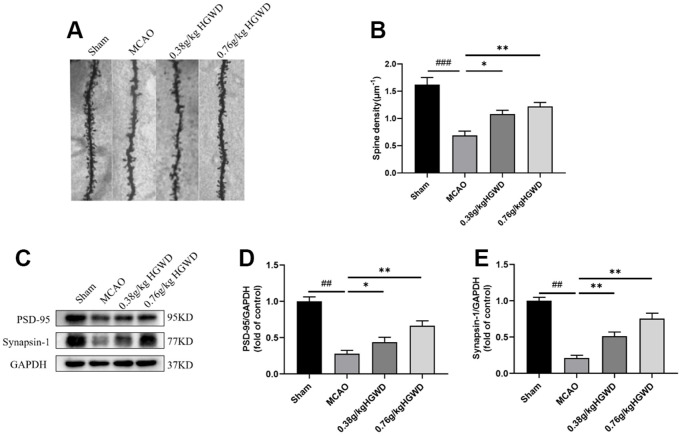
**HGWD promoted dendritic spine density and synaptic plasticity.** (**A**) Examples of dendritic spines. Scale bar=10 μm. (**B**) Density of dendritic spines. (**C**) Representative western blot bands of PSD95 and synapsin I in each group. (**D**, **E**) The quantitative analysis of PSD95 and synapsin I expression in hippocampus. ^##^*P* < 0.01 vs. control group; *P< 0.05, **P< 0.01 vs. model group.

Previous studies have reported that microglia could modify synaptic plasticity involving “find-me” and “eat-me” pathways [25]. Considering the promotion of HGWD on M2 polarization, we further tested whether HGWD produce beneficial effect on synaptic plasticity. As displayed in Figure 5A, 5B, the density of dendritic spines decreased in the MCAO group compared to the Sham group (*P* < 0.001). HGWD could dose-dependently increased the dendritic spine density when compared to the MCAO group. The expression levels of synapse marker proteins (PSD95, Synapsin I) were also determined to confirm the effect of HGWD on synaptic plasticity. The results showed that MCAO significantly decreased the protein levels of PSD95 and Synapsin 1 (*P*< 0.01, both) compared to the Sham group. The expression of PSD95 and Synapsin I were obviously upregulated in HGWD-treated rat compared to the MCAO rat.

### HGWD modified Sirt1/NF-κB/NLRP3 pathway in the hippocampus of MCAO rats

**Figure 6:**
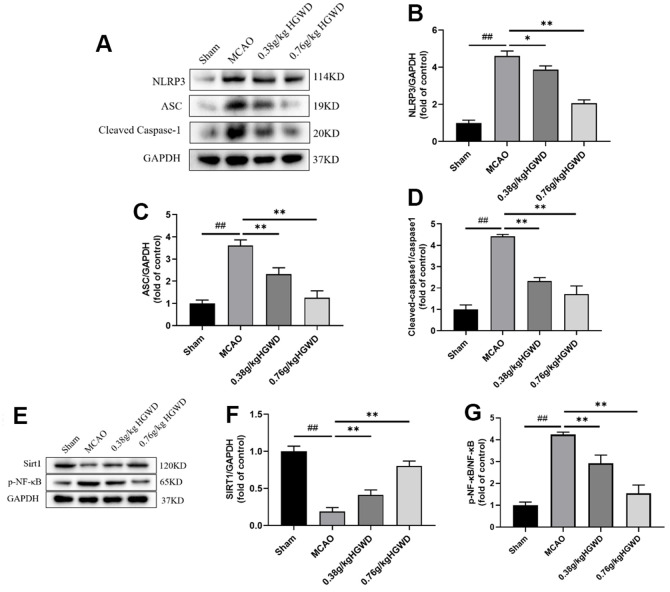
**HGWD modified Sirt1/NF-κB/NLRP3 pathway in the hippocampus of MCAO rats.** (**A**) Representative western blot bands of NLRP3, ASC and Cleaved Caspase-1 in each group. (**B**–**D**) The quantitative analysis of NLRP3, ASC and Cleaved Caspase-1 protein expression. (**E**) Representative western blot bands of Sirt1 and p-NF-κB in each group. (**F**, **G**) The quantitative analysis of Sirt1 and p-NF-κB protein expression. ^##^*P* < 0.01 vs. control group; *P< 0.05, **P< 0.01 vs. model group.

Infammasome NLRP3 plays pivotal roles in regulating microglia polarization and neuroinflammation. To study the underlying mechanism of neuroprotective action of HGWD, NLRP3 inflammasome-associated proteins in the hippocampi of rat were probed. Western blotting revealed that, in comparison with Sham group, the expression of the main components of NLRP3 inflammasome, NLRP3, ASC, and cleaved caspase-1 were enhanced by cerebral ischemia-reperfusion injury. Notably, administration of HGWD significantly mitigated the activation of NLRP3 inflammasome in MCAO rats (Figure 6A–6D).

Recently, it has been reported that NLRP3 inflammasome could be inhibited through Sirt1-dependent pathway [26]. Selective Sirt1 antagonist was reported to suppress NF-κB activity, and subsequently blunted NLRP3 inflammasome activation [27]. In order to further detect whether HGWD affected Sirt1/NF-κB pathway, the relative protein levels of Sirt1 and p-NF-κB in in the hippocampi of rat were measured (Figure 6E–6G). Consistently, HGWD-treated groups significantly enhanced Sirt1 protein but down-regulated p-NF-κB levels.

### Inhibition of Sirt1 weakened the neuroprotective effect of HGWD *in vivo*

**Figure 7:**
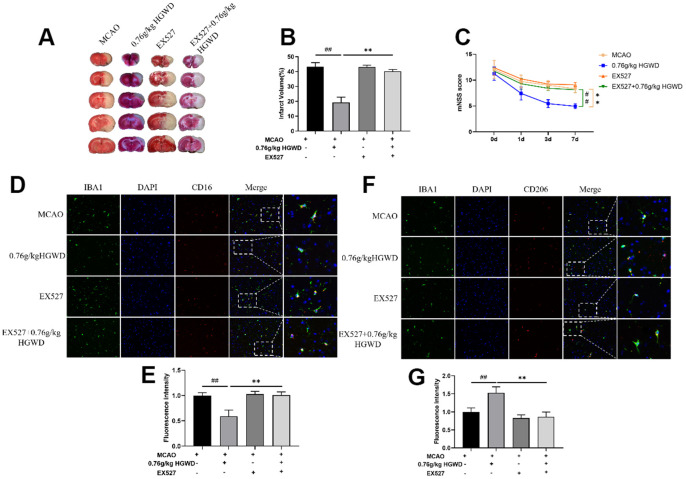
**EX527 dramatically blocked the efficacy of HGWD on neurological deficits and microglia polarization in MCAO rats.** (**A**) Representative TTC staining section. (**B**) Quantitative analysis of infarct regions. (**C**) The neurological scores (mNSS). (**D**) Representative double-immunofluorescence staining for CD16 (red) and Iba-1 (green) markers in hippocampus. (**E**) Quantification of the fluorescence intensity of CD16^+^/Iba-1^+^ cells. (**F**) Representative double-immunofluorescence staining for CD206 (red) and Iba-1 (green) markers in the hippocampus. (**G**) Quantification of fluorescence intensity of CD206^+^/Iba-1^+^ cells. Scale bar: 50 μm. ^##^*P* < 0.01 vs. control group; **P< 0.01 vs. model group.

**Figure 8:**
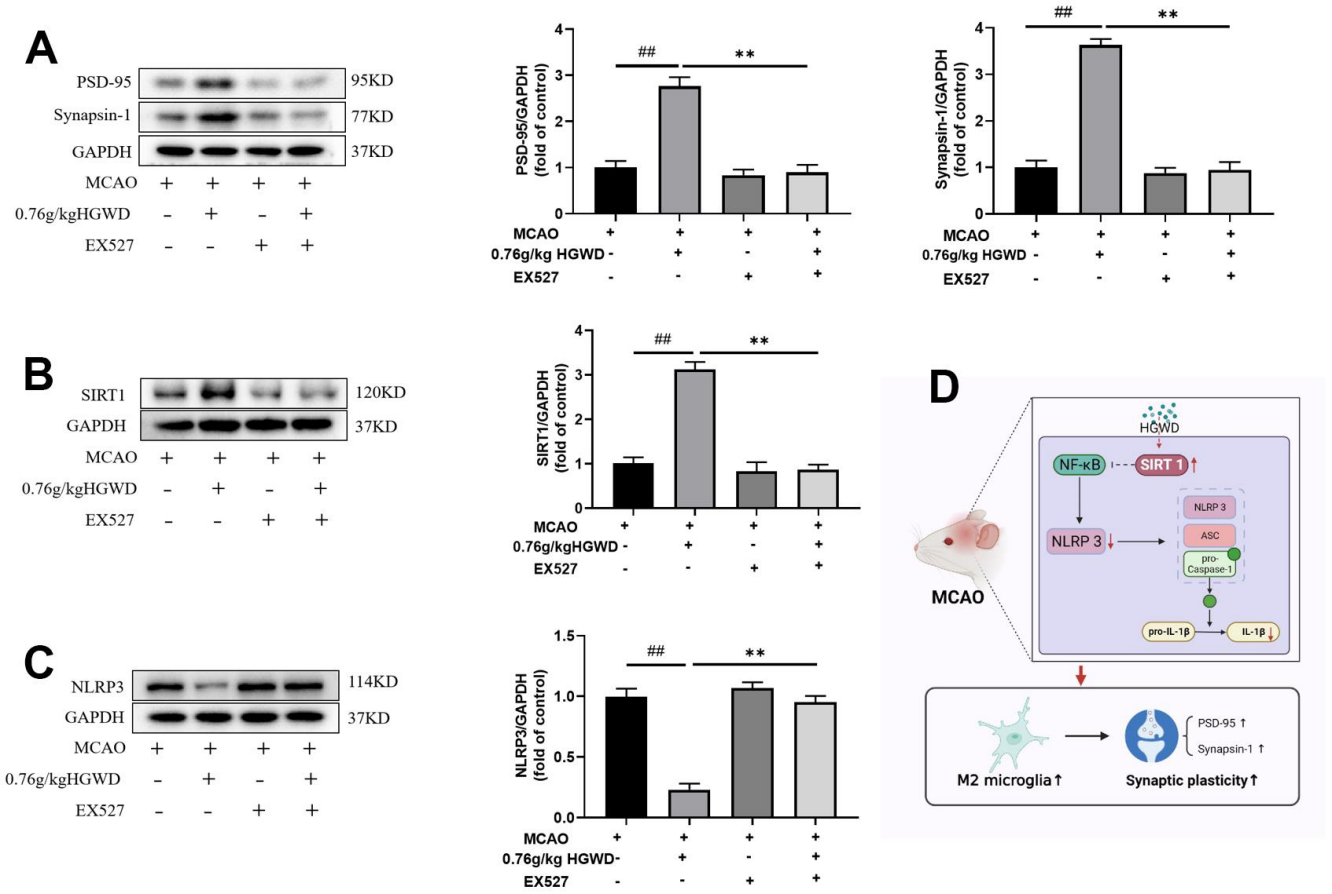
**EX527 notably weakened the modulation of HGWD on PSD95, Sirt1 and NLRP3 expression.** (**A**) PSD95 expression in each group. Left, representative western blot. Right, Quantitative analysis of PSD95. (**B**) Sirt1 expression in each group. Left, representative western blot. Right, Quantitative analysis of Sirt1. (**C**) NLRP3 expression in each group. Left, representative western blot. Right, Quantitative analysis of NLRP3. (**D**) Schematic representation of mechanisms by which HGWD protect against MCAO injury. ^##^*P* < 0.01 vs. control group; **P< 0.01 vs. model group. ^##^*P* < 0.01 vs. control group; **P< 0.01 vs. model group.

Finally, we further explored the role of Sirt1 in the therapeutic effect of HGWD. Sirt1 specific inhibitor EX527 (10mg/kg) was injected into the subarachnoid space prior to MCAO operation. As shown in Figure 7A–7C, the infarct volume and mNSS score in the MCAO+H-HGWD (0.76mg/kg) group was significantly decreased compared with MCAO group (*P* < 0.01). The effects of HGWD on reduction of infarct size and improvement of neurological functions were markedly abolished by EX527. Besides, the number of CD16+ M1 microglia decreased and CD206+ M2 microglia increased in the MCAO+H-HGWD (0.76mg/kg) group compared with that of the MCAO group (*P* < 0.01, both; Figure 7D–7G). However, there was no significant difference between the microglia M1/M2 polarization in the MCAO+EX-527+H-HGWD (0.76mg/kg) group and the MCAO group. Next, the expressions of PSD95, Sirt1 and NLRP3 were detected. Western blot revealed that, compared with the MCAO+H-HGWD group, the expression levels of PSD95, Sirt1 (*P* <0.01) were pronouncedly decreased and NLRP3 was higher (*P*<0.01) in the MCAO+EX-527+H-HGWD group (Figure 8A–8C). The above data indicated that inhibiting SIRT1 could notably weakened the therapeutic effect of HGWD against MCAO.

## DISCUSSION

Cerebral infarction refers to ischemic necrosis or softening of tissues caused by ischemia and hypoxia due to the obstruction of the blood supply to the brain. Cerebral infarction has attracted much attention in the medical field because of its high rate of disability, mortality and recurrence. Pharmacological studies have shown that traditional Chinese medicine (TCM) can inhibit platelet aggregation, promote blood circulation, defend against ischemia reperfusion injury, and increase the tolerance of ischemic brain tissue to hypoxia. In clinical practice, TCM application in the treatment of cerebral infarction significantly improve the clinical symptoms of patients, restore the function of hemiplegic limbs, and delay the progression of stroke. According to TCM theory, stroke is caused by Qi deficiency and blood stasis. TCM which can replenish Qi and promote blood circulation to remove blood stasis can be used to treat stroke. HGWD is a classic prescription from the Golden Chamber, and has long been used to treat blood arthralgia due to Qi deficiency and blood stagnation. Numerous clinical practices show that HGWD exerts good therapeutic effects for ischemic stroke. However, the neuroprotective mechanism of HGWD remains to be clarified. In this work, we confirmed that HGWD can significantly reduce the cerebral infarction area of MCAO rats, improve the pathological damage of brain tissue, and inhibit the release of inflammatory factors and oxidative stress (Figures 2, 3), which is consistent with the results of previous studies [28]. Additionally, further molecular mechanism studies showed that HGWD promoted microglia polarization to M2 phenotype, and enhanced synaptic plasticity by activating Sirt1/NF-κB/NLRP3 pathway, thus playing a protective role against cerebral ischemic injury.

Neuroinflammation regulated by microglia has been identified to be a pivotal factor in the development of central nervous system (CNS) diseases. Overactivated microglia would stimulate the release of pro-inflammatory cytokines into the neuronal microenvironment, thus preventing neuronal regeneration and causing neuronal damage [29]. Microglia have a dual polarization state, pro-inflammatory M1 and anti-inflammatory M2 phenotypes. CD16 and CD206 are the phenotypic markers of microglia M1 and M2 respectively. Actually, M1/M2 transformation of microglia has been observed in various CNS disease including stroke [30]. M2 microglia can engulf damaged neuron debris, inhibit excessive inflammatory response, and promote tissue repair and neuronal regeneration. Regulating microglia polarization from M1 toward M2 after cerebral ischemia is beneficial for the improvement of brain impairment and functional recovery [31]. In our study, the colocalization of CD16^+^/Iba1^+^ and CD206^+^/Iba1^+^ in the ischemic penumbra of MCAO rats was determined. Iba1 is considered to be a common marker of microglia. The results showed that HGWD markedly decreased the number of CD16^+^/Iba1^+^ positive cells, but increased the number of CD206+/Iba1+ positive cells (Figure 4B, 4C). Besides, HGWD downregulated the expression of iNOS and CD16 mRNA, and upregulated the level of CD206 and Arg-1 mRNA (Figure 4A). These results indicated that HGWD promoted microglia polarization to the M2 phenotype in the ischemic penumbra of MCAO/R rats.

Synaptic plasticity will be severely damaged after ischemia [32]. Microglia have been found to play a key role in the regulation of synaptic function in central nervous system diseases. *In vivo* observation by two-photon microscopy showed that the contact time and frequency between microglia and synapses significantly increased after cerebral ischemia, indicating that microglia were involved in the remodeling of neural circuits after cerebral ischemia [33]. In AD mice and multiple sclerosis models, microglia can induce synaptic loss and cognitive impairment through C1q-C3 signaling axis-mediated synaptic phagocytosis [34, 35]. Our experiment has confirmed that HGWD can promote the transformation of microglia from M1 to M2. Next, we investigated whether HGWD could affect neural synaptic plasticity. The experimental results showed that, compared with MCAO model group, HGWD group could significantly enhance the synaptic density and expression of synaptic marker proteins (PSD95, Synapsin I), suggesting that HGWD could improve the synaptic plasticity damage caused by cerebral ischemia (Figure 5A–5E).

Next, we further explored the molecular mechanism mediating the regulation of HGWD on microglia polarization. Recent studies have confirmed the protection role of Sirt1 on the improvement of cerebral ischemia/reperfusion injury [36, 37]. As a member of the deacetylase family, Sirt1 also plays an important role in regulating microglia/macrophage polarization and alleviating inflammatory damage [38, 39]. Activation of Sirt1 has been considered as a neuroprotective strategy. Sirt1 agonist can ameliorate subarachnoid hemorrhage injury via promoting M2 microglia polarization [40]. Sirt1 activation also can inhibit nuclear transposition of NF-κB, reduce the release of proinflammatory cytokine such as TNF-α and IL-6, and then alleviate the inflammatory response [41]. In addition, Sirt1 could inhibit inflammasome activation through NF-κB suppression. NF-κB can activate NLRP3 inflammasome, promote the production of pro-IL-1β and pro-IL-18, and aggravate the inflammatory response and induce caspase-1 dependent pyroptotic cell death [42]. Actually, several bioactive components isolated from the major crude drugs of HGWD, have been previously demonstrated to have a regulatory effect on Sirt1. Cycloastragenol, the active form of astragaloside IV from Radix astragali, upregulated Sirt1 expression and then suppressed neuroinflammation after brain ischemia [43]. Isoflavonoid formononetin was reported to attenuate renal tubular injury by upregulating Sirt1/PGC-1α pathway in diabetic nephropathy [44]. Paeonol form Radix paeoniae alba was also reported to inhibit inflammatory response in chondrocytes by upregulating Sirt1 [45]. Besides, bioactive chemicals in HGWD including calycosin can modulate NLRP3 and inhibit pyroptotic cell death [46]. In this study, we found that HGWD increased the protein level of Sirt1, and reduced the expression of p-NF-κB, NLRP3, ASC, and Cleaved Caspase-1 (Figure 6). When co-administration with Sirt1 specific inhibitor EX527, the improvement effect of HGWD on brain injury, microglia polarization, and synaptic plasticity were significantly abolished, suggesting that Sirt1 might be an important target of HGWD (Figures 7, 8). HGWD may alleviate cerebral ischemia injury via Sirt1/NF-κB/NLRP3 inflammatory signaling pathway (Figure 8D). In our follow-up study, we would further confirm the role of Sirt1 in the neuroprotective effect of HGWD using knockout mice.

## CONCLUSIONS

In summary, our study demonstrated *in vivo* that, HGWD could effectively ameliorate cerebral injury, reduce inflammatory damage, shift microglia polarization towards M2 and enhance synaptic plasticity. Besides, as to our knowledge, this work also demonstrated for the first time, that HGWD promoted microglial M2 polarization and synaptic plasticity at least partially via activating Sirt1/NF-κB/NLRP3 signaling pathway. HGWD exhibits promising therapeutic effect for ischemic stroke. Further research before clinical application are needed to reveal the bioactive constituents in HGWD and its more detailed mechanism.

## References

[1] Wang YJ, Li ZX, Gu HQ, Zhai Y, Jiang Y, Zhao XQ, Wang YL, Yang X, Wang CJ, Meng X, Li H, Liu LP, Jing J, et al, and China Stroke Statistics 2019 Writing Committee. China Stroke Statistics 2019: A Report From the National Center for Healthcare Quality Management in Neurological Diseases, China National Clinical Research Center for Neurological Diseases, the Chinese Stroke Association, National Center for Chronic and Non-communicable Disease Control and Prevention, Chinese Center for Disease Control and Prevention and Institute for Global Neuroscience and Stroke Collaborations. Stroke Vasc Neurol. 2020; 5:211–39. 10.1136/svn-2020-000457 32826385 PMC7548521

[2] Mizuma A, You JS, Yenari MA. Targeting Reperfusion Injury in the Age of Mechanical Thrombectomy. Stroke. 2018; 49:1796–802. 10.1161/STROKEAHA.117.017286 29760275 PMC6019565

[3] Mbabuike N, Gassie K, Brown B, Miller DA, Tawk RG. Revascularization of tandem occlusions in acute ischemic stroke: review of the literature and illustrative case. Neurosurg Focus. 2017; 42:E15. 10.3171/2017.1.FOCUS16521 28366063

[4] Eldahshan W, Fagan SC, Ergul A. Inflammation within the neurovascular unit: Focus on microglia for stroke injury and recovery. Pharmacol Res. 2019; 147:104349. 10.1016/j.phrs.2019.104349 31315064 PMC6954670

[5] Lambertsen KL, Finsen B, Clausen BH. Post-stroke inflammation-target or tool for therapy? Acta Neuropathol. 2019; 137:693–714. 10.1007/s00401-018-1930-z 30483945 PMC6482288

[6] Poh L, Kang SW, Baik SH, Ng GY, She DT, Balaganapathy P, Dheen ST, Magnus T, Gelderblom M, Sobey CG, Koo EH, Fann DY, Arumugam TV. Evidence that NLRC4 inflammasome mediates apoptotic and pyroptotic microglial death following ischemic stroke. Brain Behav Immun. 2019; 75:34–47. 10.1016/j.bbi.2018.09.001 30195027

[7] Li Q, Barres BA. Microglia and macrophages in brain homeostasis and disease. Nat Rev Immunol. 2018; 18:225–42. 10.1038/nri.2017.125 29151590

[8] Liao S, Wu J, Liu R, Wang S, Luo J, Yang Y, Qin Y, Li T, Zheng X, Song J, Zhao X, Xiao C, Zhang Y, et al. A novel compound DBZ ameliorates neuroinflammation in LPS-stimulated microglia and ischemic stroke rats: Role of Akt(Ser473)/GSK3β(Ser9)-mediated Nrf2 activation. Redox Biol. 2020; 36:101644. 10.1016/j.redox.2020.101644 32863210 PMC7371982

[9] Qin C, Zhou LQ, Ma XT, Hu ZW, Yang S, Chen M, Bosco DB, Wu LJ, Tian DS. Dual Functions of Microglia in Ischemic Stroke. Neurosci Bull. 2019; 35:921–33. 10.1007/s12264-019-00388-3 31062335 PMC6754485

[10] Yang X, Xu S, Qian Y, Xiao Q. Resveratrol regulates microglia M1/M2 polarization via PGC-1α in conditions of neuroinflammatory injury. Brain Behav Immun. 2017; 64:162–72. 10.1016/j.bbi.2017.03.003 28268115

[11] Mangan MSJ, Olhava EJ, Roush WR, Seidel HM, Glick GD, Latz E. Targeting the NLRP3 inflammasome in inflammatory diseases. Nat Rev Drug Discov. 2018; 17:588–606. 10.1038/nrd.2018.97 30026524

[12] Zhou K, Shi L, Wang Y, Chen S, Zhang J. Recent Advances of the NLRP3 Inflammasome in Central Nervous System Disorders. J Immunol Res. 2016; 2016:9238290. 10.1155/2016/9238290 27652274 PMC5019917

[13] Ye Y, Jin T, Zhang X, Zeng Z, Ye B, Wang J, Zhong Y, Xiong X, Gu L. Meisoindigo Protects Against Focal Cerebral Ischemia-Reperfusion Injury by Inhibiting NLRP3 Inflammasome Activation and Regulating Microglia/Macrophage Polarization via TLR4/NF-κB Signaling Pathway. Front Cell Neurosci. 2019; 13:553. 10.3389/fncel.2019.00553 31920554 PMC6930809

[14] Franke M, Bieber M, Kraft P, Weber AN, Stoll G, Schuhmann MK. The NLRP3 inflammasome drives inflammation in ischemia/reperfusion injury after transient middle cerebral artery occlusion in mice. Brain Behav Immun. 2021; 92:223–33. 10.1016/j.bbi.2020.12.009 33307174

[15] Wang S, Lin Y, Yuan X, Li F, Guo L, Wu B. REV-ERBα integrates colon clock with experimental colitis through regulation of NF-κB/NLRP3 axis. Nat Commun. 2018; 9:4246. 10.1038/s41467-018-06568-5 30315268 PMC6185905

[16] Li Y, Wang P, Yang X, Wang W, Zhang J, He Y, Zhang W, Jing T, Wang B, Lin R. SIRT1 inhibits inflammatory response partly through regulation of NLRP3 inflammasome in vascular endothelial cells. Mol Immunol. 2016; 77:148–56. 10.1016/j.molimm.2016.07.018 27505710

[17] Jiang W, Zhang FJ, Zao BH. Effect of Huangqi Guizhi Wuwu Decoction on patients with convalescent cerebral infarction. World J Traditional Chinese Med. 2017; 12:1555–9.

[18] Pang B, Zhao TY, Zhao LH, Wan F, Ye R, Zhou Q, Tian F, Tong XL. Huangqi Guizhi Wuwu Decoction for treating diabetic peripheral neuropathy: a meta-analysis of 16 randomized controlled trials. Neural Regen Res. 2016; 11:1347–58. 10.4103/1673-5374.189202 27651785 PMC5020836

[19] Wei XM, Chen XF, Shu P, Jiang ZW, Wu XY, Zou X, Chen K, Shen B, Hu WW, Lu W, Shen WX, Li L, Wang JY, et al. Study on efficacy and safety of Huangqi Guizhi Wuwu decoction treatment for oxaliplatin induced peripheral neurotoxicity: A protocol for a randomized, controlled, double-blind, multicenter trial. Medicine (Baltimore). 2020; 99:e19923. 10.1097/MD.0000000000019923 32481364 PMC12245263

[20] Yin YX, Ren HQ. Treatment of 46 cases of sequelae of cerebral infarction with Huangqi Guizhi Wuwu Decoction. Inner Mongolia Traditional Chinese Med. 2017; 36:11–4.

[21] Zhang Y, Qiao L, Xu W, Wang X, Li H, Xu W, Chu K, Lin Y. Paeoniflorin Attenuates Cerebral Ischemia-Induced Injury by Regulating Ca^2+^/CaMKII/CREB Signaling Pathway. Molecules. 2017; 22:359. 10.3390/molecules22030359 28264448 PMC6155252

[22] Yan X, Yu A, Zheng H, Wang S, He Y, Wang L. Calycosin-7-*O*-*β*-*D*-glucoside Attenuates OGD/R-Induced Damage by Preventing Oxidative Stress and Neuronal Apoptosis via the SIRT1/FOXO1/PGC-1*α* Pathway in HT22 Cells. Neural Plast. 2019; 2019:8798069. 10.1155/2019/8798069 31885537 PMC6915014

[23] Hsu CC, Kuo TW, Liu WP, Chang CP, Lin HJ. Calycosin Preserves BDNF/TrkB Signaling and Reduces Post-Stroke Neurological Injury after Cerebral Ischemia by Reducing Accumulation of Hypertrophic and TNF-α-Containing Microglia in Rats. J Neuroimmune Pharmacol. 2020; 15:326–39. 10.1007/s11481-019-09903-9 31927682

[24] Shi YH, Zhang XL, Ying PJ, Wu ZQ, Lin LL, Chen W, Zheng GQ, Zhu WZ. Neuroprotective Effect of Astragaloside IV on Cerebral Ischemia/Reperfusion Injury Rats Through Sirt1/Mapt Pathway. Front Pharmacol. 2021; 12:639898. 10.3389/fphar.2021.639898 33841157 PMC8033022

[25] Saxena S, Kruys V, Vamecq J, Maze M. The Role of Microglia in Perioperative Neuroinflammation and Neurocognitive Disorders. Front Aging Neurosci. 2021; 13:671499. 10.3389/fnagi.2021.671499 34122048 PMC8193130

[26] Zhang XS, Lu Y, Li W, Tao T, Wang WH, Gao S, Zhou Y, Guo YT, Liu C, Zhuang Z, Hang CH, Li W. Cerebroprotection by dioscin after experimental subarachnoid haemorrhage via inhibiting NLRP3 inflammasome through SIRT1-dependent pathway. Br J Pharmacol. 2021; 178:3648–66. 10.1111/bph.15507 33904167

[27] Zhou Y, Wang S, Wan T, Huang Y, Pang N, Jiang X, Gu Y, Zhang Z, Luo J, Yang L. Cyanidin-3-O-β-glucoside inactivates NLRP3 inflammasome and alleviates alcoholic steatohepatitis via SirT1/NF-κB signaling pathway. Free Radic Biol Med. 2020; 160:334–41. 10.1016/j.freeradbiomed.2020.08.006 32805401

[28] Ling JY, Wang Q, Fan HZ. Huangqi Guizhi Wuwu decoction ameliorates focal cerebral ischemia-reperfusion injury in rats: pharmacological and metabolomics evidences. J Nanjing Univ Tradit Chin Med. 2021; 37:920–9.

[29] Subhramanyam CS, Wang C, Hu Q, Dheen ST. Microglia-mediated neuroinflammation in neurodegenerative diseases. Semin Cell Dev Biol. 2019; 94:112–20. 10.1016/j.semcdb.2019.05.004 31077796

[30] Mesquida-Veny F, Del Río JA, Hervera A. Macrophagic and microglial complexity after neuronal injury. Prog Neurobiol. 2021; 200:101970. 10.1016/j.pneurobio.2020.101970 33358752

[31] Ma DC, Zhang NN, Zhang YN, Chen HS. Salvianolic Acids for Injection alleviates cerebral ischemia/reperfusion injury by switching M1/M2 phenotypes and inhibiting NLRP3 inflammasome/pyroptosis axis in microglia *in vivo* and *in vitro*. J Ethnopharmacol. 2021; 270:113776. 10.1016/j.jep.2021.113776 33421597

[32] Yao Y, Wang F, Yang X, Zang D, Yang J, Wang Z. Bombesin attenuated ischemia-induced spatial cognitive and synaptic plasticity impairment associated with oxidative damage. Biomed Pharmacother. 2018; 103:87–93. 10.1016/j.biopha.2018.03.155 29635132

[33] Wake H, Moorhouse AJ, Jinno S, Kohsaka S, Nabekura J. Resting microglia directly monitor the functional state of synapses *in vivo* and determine the fate of ischemic terminals. J Neurosci. 2009; 29:3974–80. 10.1523/JNEUROSCI.4363-08.2009 19339593 PMC6665392

[34] Hong S, Beja-Glasser VF, Nfonoyim BM, Frouin A, Li S, Ramakrishnan S, Merry KM, Shi Q, Rosenthal A, Barres BA, Lemere CA, Selkoe DJ, Stevens B. Complement and microglia mediate early synapse loss in Alzheimer mouse models. Science. 2016; 352:712–6. 10.1126/science.aad8373 27033548 PMC5094372

[35] Michailidou I, Willems JG, Kooi EJ, van Eden C, Gold SM, Geurts JJ, Baas F, Huitinga I, Ramaglia V. Complement C1q-C3-associated synaptic changes in multiple sclerosis hippocampus. Ann Neurol. 2015; 77:1007–26. 10.1002/ana.24398 25727254

[36] Yan L, Zhu T. Effects of rosuvastatin on neuronal apoptosis in cerebral ischemic stroke rats via Sirt1/NF-kappa B signaling pathway. Eur Rev Med Pharmacol Sci. 2019; 23:5449–55. 10.26355/eurrev_201906_18214 31298398

[37] Luo Y, Chen H, Tsoi B, Wang Q, Shen J. Danggui-Shaoyao-San (DSS) Ameliorates Cerebral Ischemia-Reperfusion Injury via Activating SIRT1 Signaling and Inhibiting NADPH Oxidases. Front Pharmacol. 2021; 12:653795. 10.3389/fphar.2021.653795 33935765 PMC8082392

[38] Zhou H, Xu Z, Liao X, Tang S, Li N, Hou S. Low Expression of YTH Domain-Containing 1 Promotes Microglial M1 Polarization by Reducing the Stability of Sirtuin 1 mRNA. Front Cell Neurosci. 2021; 15:774305. 10.3389/fncel.2021.774305 34975410 PMC8714917

[39] Qin X, Chen J, Zhang G, Li C, Zhu J, Xue H, Li J, Guan T, Zheng H, Liu Y, Cai H. Hydroxysafflor Yellow A Exerts Anti-Inflammatory Effects Mediated by SIRT1 in Lipopolysaccharide-Induced Microglia Activation. Front Pharmacol. 2020; 11:1315. 10.3389/fphar.2020.01315 33041785 PMC7517830

[40] Xia DY, Yuan JL, Jiang XC, Qi M, Lai NS, Wu LY, Zhang XS. SIRT1 Promotes M2 Microglia Polarization via Reducing ROS-Mediated NLRP3 Inflammasome Signaling After Subarachnoid Hemorrhage. Front Immunol. 2021; 12:770744. 10.3389/fimmu.2021.770744 34899720 PMC8653696

[41] de Gregorio E, Colell A, Morales A, Marí M. Relevance of SIRT1-NF-κB Axis as Therapeutic Target to Ameliorate Inflammation in Liver Disease. Int J Mol Sci. 2020; 21:3858. 10.3390/ijms21113858 32485811 PMC7312021

[42] Chi W, Chen H, Li F, Zhu Y, Yin W, Zhuo Y. HMGB1 promotes the activation of NLRP3 and caspase-8 inflammasomes via NF-κB pathway in acute glaucoma. J Neuroinflammation. 2015; 12:137. 10.1186/s12974-015-0360-2 26224068 PMC4518626

[43] Li M, Li SC, Dou BK, Zou YX, Han HZ, Liu DX, Ke ZJ, Wang ZF. Cycloastragenol upregulates SIRT1 expression, attenuates apoptosis and suppresses neuroinflammation after brain ischemia. Acta Pharmacol Sin. 2020; 41:1025–32. 10.1038/s41401-020-0386-6 32203080 PMC7471431

[44] Huang Q, Chen H, Yin K, Shen Y, Lin K, Guo X, Zhang X, Wang N, Xin W, Xu Y, Gui D. Formononetin Attenuates Renal Tubular Injury and Mitochondrial Damage in Diabetic Nephropathy Partly via Regulating Sirt1/PGC-1α Pathway. Front Pharmacol. 2022; 13:901234. 10.3389/fphar.2022.901234 35645821 PMC9133725

[45] Shang P, Liu Y, Jia J. Paeonol inhibits inflammatory response and protects chondrocytes by upregulating sirtuin 1. Can J Physiol Pharmacol. 2022; 100:283–90. 10.1139/cjpp-2021-0319 35235465

[46] Yosri H, El-Kashef DH, El-Sherbiny M, Said E, Salem HA. Calycosin modulates NLRP3 and TXNIP-mediated pyroptotic signaling and attenuates diabetic nephropathy progression in diabetic rats; An insight. Biomed Pharmacother. 2022; 155:113758. 10.1016/j.biopha.2022.113758 36271546

